# Identification of key genes of the ccRCC subtype with poor prognosis

**DOI:** 10.1038/s41598-022-18620-y

**Published:** 2022-08-26

**Authors:** Grigory Andreevich Puzanov

**Affiliations:** 1 Laboratory of Intracellular Signaling in Health and Disease, Engelhardt Institute of Molecular Biology, Russian Academy of Sciences, Vavilova Str., 32, Moscow, Russia 119991; 2 Laboratory of Bioinformatics, Faculty of Computer Science, National Research University Higher School of Economics, Moscow, Russia

**Keywords:** Cancer genomics, Renal cell carcinoma, Cancer microenvironment, Tumour heterogeneity, Tumour biomarkers, Functional clustering

## Abstract

Clear cell renal carcinoma has been reported in many research studies as a rather heterogeneous disease. Identification of different subtypes and their molecular characteristics can help in choosing a more effective treatment and predicting a response to it. In this study, using multi-omics clustering of RNA-Seq data of patients with clear cell renal carcinoma from TCGA. Specific genes were identified for the most aggressive ccRCC subtype associated with metastasis and a subtype associated with a more favorable course of the disease. Among them were genes associated with blood clotting (*FGA*, *FGG*) and genes associated with changes in the immune characteristics of a tumor (*ENAM*, *IGFBP1*, *IL6*). In addition, an association of hub genes of poor survival ccRCC subtype with the levels of infiltration of endothelial cells, hematopoietic stem cells, T cells NK and mast cells was revealed. It was shown that *MFI2*, *CP*, *FGA*, and *FGG* expression can predict the response to sunitinib, while the *APOB*, *ENAM*, *IGFBP1*, and *MFI2* expression predict the response to nivolumab. The results obtained provide insight into the genetic characteristics underlying the aggressive subtype of ccRCC and may help develop new approaches to the treatment of this disease.

## Introduction

Clear cell renal carcinoma (ccRCC) is the most common subtype of renal cancer^1^. The 5-year survival of ccRCC patients is about 60–70%^2^. The available data show an increase in the number of new cases of ccRCC over the past decades^2^. Despite the extensive data accumulated recently, the identification of reliable genes that make it possible to predict and select therapy for ccRCC is still an urgent problem.

To date, abundant evidence suggests that ccRCC is a rather heterogeneous type of cancer^3^. In particular, subtypes of metastatic ccRCC have been identified based on their response to sunitinib^4^. Recently, different subtypes of ccRCC have been identified based on the activity of transcription factors^5^. These data show that within the histologic type, ccRCC group of samples can be distinguished by the general unique characteristics.

Common TNM classification of samples is often insufficient to prescribe a suitable targeted treatment^6^. It was shown that gene expression depends on the stages of ccRCC, and different stages of ccRCC are characterized by different aberrant regulated pathways, including the coagulation cascade^7^.

Early identification of the most aggressive tumors could significantly improve the treatment of ccRCC. The identification of key molecular changes specific to such malignant subtypes can help in the development of effective personalized therapies. Thus, the aim of this work is to reveal major subtypes of ccRCC tumors and to identify key genetic characteristics of these subtypes.

## Results

### Clustering of ccRCC samples revealed three subtypes with different survival rates

Clustering of ccRCC samples from the TCGA database was carried out by the k-means method with 100 replicates. For the clustering of samples, k = 3 was chosen, since it was previously reported that clustering stability increases from k = 2 to k = 3, but not at k > 3 for the ccRCC samples^3^. For analysis, I selected tumor samples for which no radiotherapy or additional pharmacotherapy was performed. As a result, 456 samples were divided into two large clusters (n = 299 and n = 126) and one smaller (n = 31).

**Figure 1:**
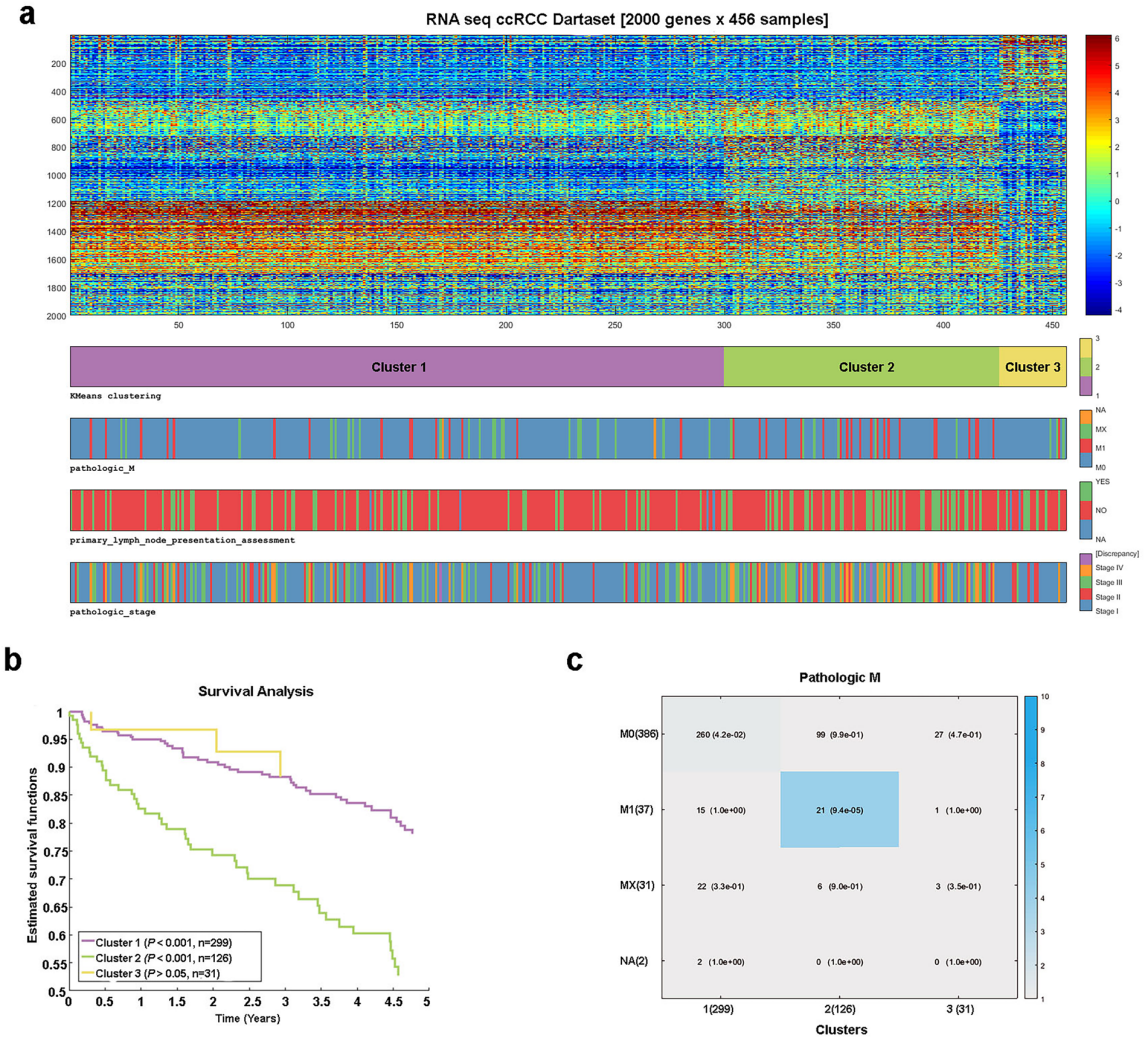
Clustering by the k-means method of ccRCC samples from the TCGA database **(a)**. The heat map shows the distribution of 456 samples across three clusters (matrix columns) and the distribution of the 2000 most variable genes in five gene clusters (matrix rows). Survival analysis for the three resulting clusters (**b**). The *P*-value shows the difference between the three groups according to the Logrank test. Distribution of samples with metastases depending on the cluster (**c**). The blue gradient increases towards a more significant *P*-value. The difference was considered significant at the *P* < 0.05. In Cluster 2, samples with the presence of metastases prevail, and in Cluster 1 with the absence of metastases.

Survival analysis revealed that Cluster 2 was associated with worse overall survival, while Cluster 1 was associated with better overall patient survival (Fig. 1b). At the same time, in Cluster 2, samples with late stages (III and IV) as well as samples with metastases prevail (Fig. 1c). While in Cluster 1, samples with stage I prevail (Fig. S1b). In addition, the available data suggests patients from Cluster 1 respond better to treatment than patients from Cluster 2 (Fig. S1c,d).

For gene clustering, I selected 2000 genes with the most highly variable expression. As a result, the genes were divided into 5 clusters (Fig. 1a). At the same time, the analysis of gene ontology shows a pronounced cluster associated with ion transport and pH maintenance, an immune response cluster, a cluster associated with an acute-phase response, a cluster associated with transmembrane organic anion transport, and a cluster associated with cell adhesion and organization of the extracellular matrix (Fig. S2). Thus, ccRCC subtypes with different survival rates and different responses to treatment were identified.

### Identification of hub genes specific for the poor prognostic subtype

**Figure 2:**
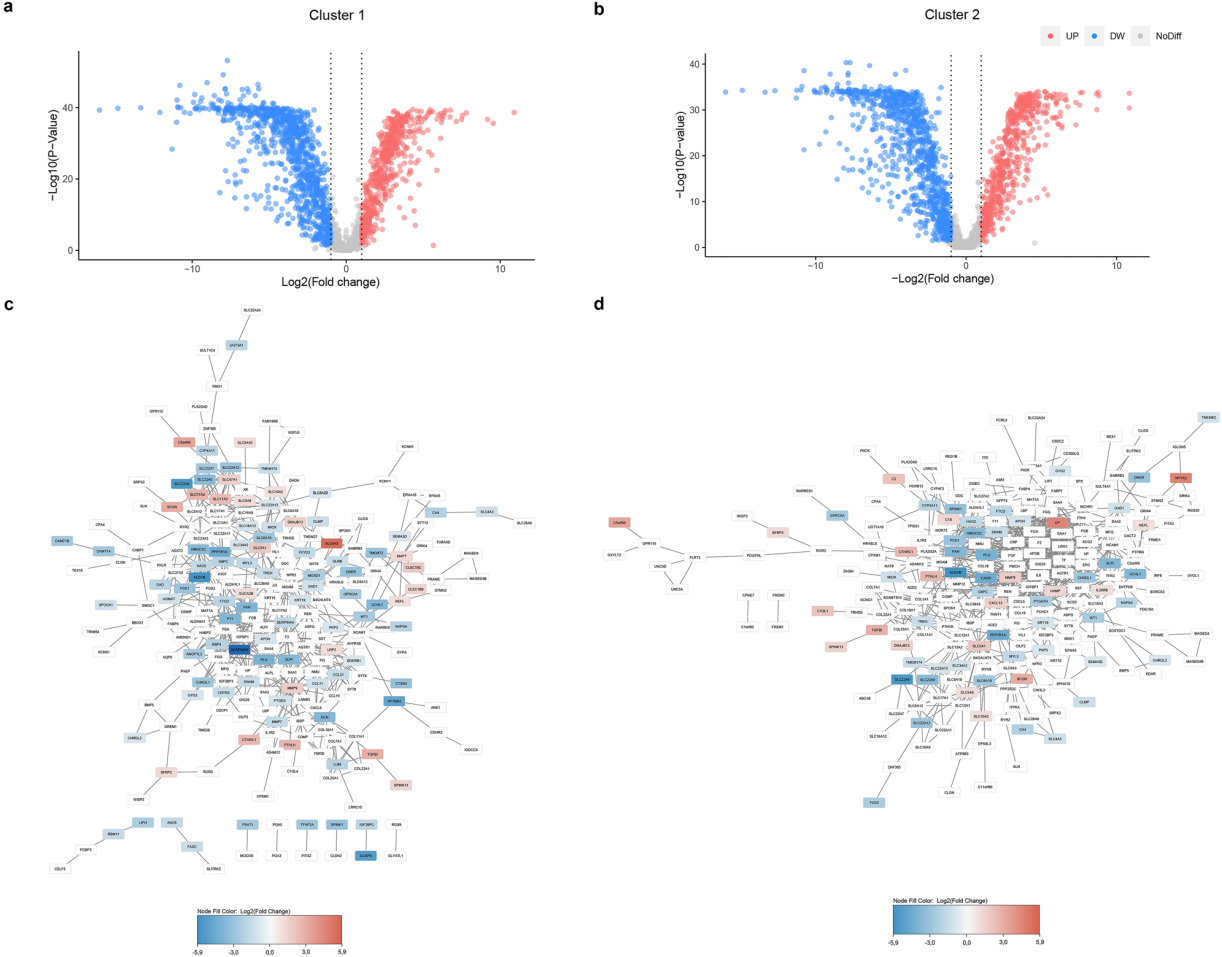
Identification of marker genes for Cluster 1 and Cluster 2 (**a**) using the rank sum test. Scheme of PPI taking into account changes in gene expression (**b**). Volcano plot of DEGs for Cluster 1 (**a**); volcano plot of DEGs for Cluster 2 (**b**). The dotted line shows the twofold change in expression clipping; red shows an increase in expression, blue a decrease in gene expression; PPI for Cluster 1 (**c**); PPI for Cluster 2 (**d**); the gradient from blue to red shows the change from lower to higher gene expression in ccRCC.

For the detected clusters, I identified differentially expressed genes using the rank sum test (Fig. 2a,b). The difference was considered significant at *P* < 0.05, and identified 310 genes for Cluster 2 and 315 genes for Cluster 1. Then, a network of protein–protein interactions (PPI) of Cluster 1 and Cluster 2 was built, taking into account changes in gene expression in ccRCC (Fig. 2c,d).

**Figure 3:**
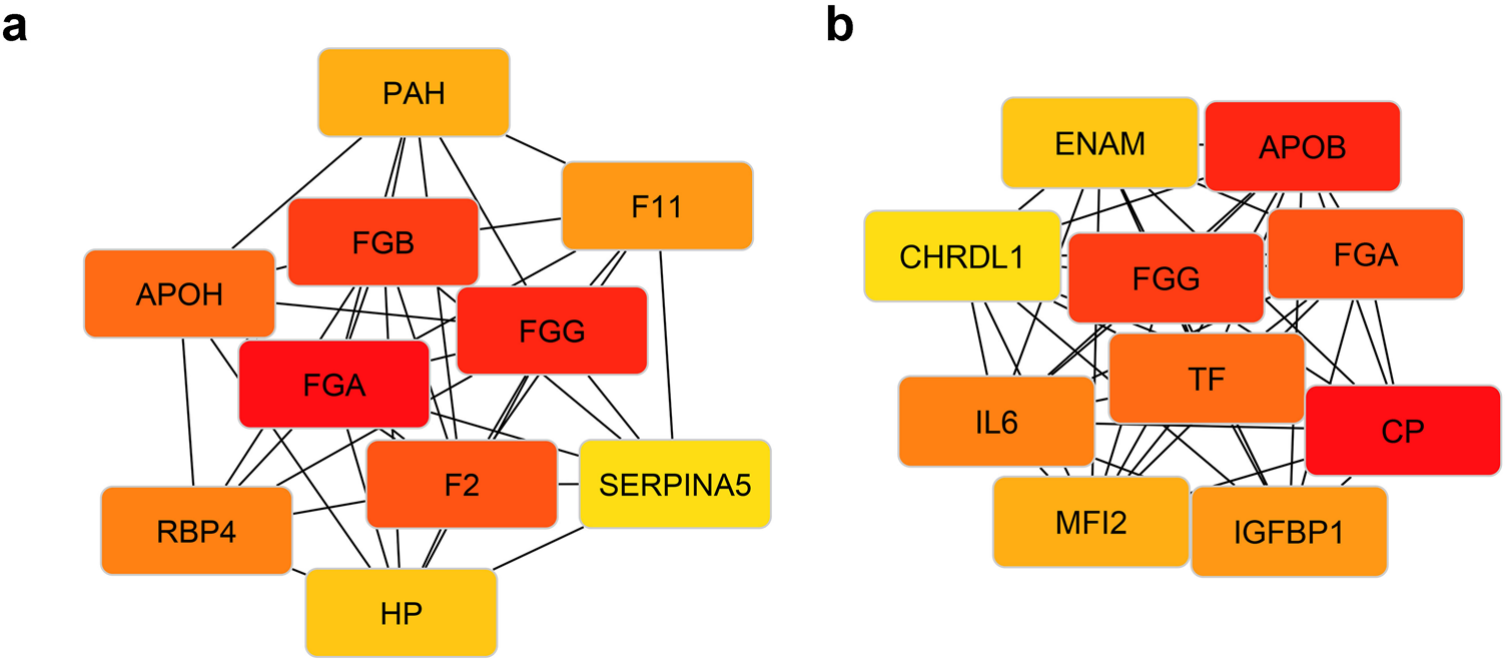
Top 10 highest score hub genes for Cluster 1 (**a**) and Cluster 2 (**b**). The edges of the graph show interactions between nodes. Red color corresponds to a higher MCC score, and thus of greater importance in the network of interactions. Yellow color corresponds to a lower MCC score, and thus of lower importance in the network of interactions.

For Clusters 1 and 2, I calculated the top 10 hub genes (Fig. 3a,b). The Maximal Clique Centrality (MCC) algorithm was used, which was reported as the most effective method to find hub genes^8^. Clustering with k = 4 for the cluster with poorest survival revealed the same 10 hub genes: *APOB*, *IGFBP1*, *CP*, *ENAM*, *MFI2*, *CHRDL1*, *IL6*, *TF*, *FGA*, *FGG*. Interestingly, among the hub genes of Cluster 1 was the *SERPINA5* gene, one of the most decreased in ccRCC (Fig. 2d). *CP* was also revealed as a hub gene for Cluster 2, which had one of the most increased expressions in ccRCC (Fig. 2c). The genes encoding the fibrinogen alpha chain (*FGA*) and fibrinogen gamma chain (*FGG*) have been identified as key for both clusters. Moreover, Cluster 2 is characterized by higher expression of these genes (Fig. 3). Gene set enrichment analysis revealed that Cluster 2 genes are associated with regulation of insulin-like growth factor, endoplasmic reticulum, and protein post-translational modifications (Fig. S3b). For Cluster 1, there is an association with fibrinolysis, hemopexin, and negative regulation of coagulation (Fig. S3a). Interestingly, for the expression of most of the detected genes, a clear bimodal or trimodal distribution was observed, which also indicates the presence of subpopulations in the sample under study (Fig. S4)^9^.

### Hub genes of the PPI network of the aggressive subtype are associated with a poor prognosis of ccRCC

**Figure 4:**
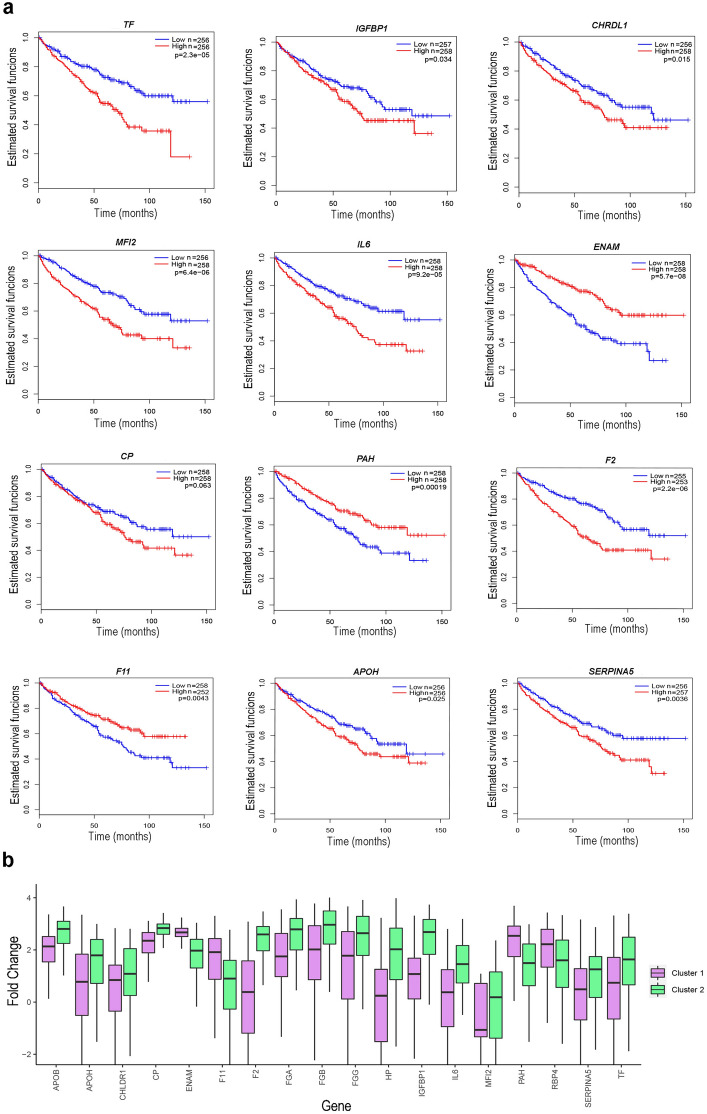
Survival curves by Kaplan–Meier method of ccRCC samples for hub genes of both clusters (**a**). Red indicates the group with higher gene expression, blue indicates the group with lower gene expression. The *P*-value of the Logrank test shows the difference in the two groups. *P* < 0.05 were considered significant. Changes in the expression levels of hub genes in the ccRCC samples (**b**). The rectangles represent interquartile ranges from 25 to 75 percentiles; the horizontal lines within the rectangles represent the medians. Vertical lines indicate the maximum and minimum values. *P* < 0.001 in each case (Wilcoxon Rank Sum Test).

Survival analysis for hub genes revealed increased expression of 5 out of 10 genes in Cluster 2 is associated with poor survival (Fig. 4a). The same cannot be said about the Cluster 1 hub genes. Gene expression varies in Cluster 1 and Cluster 2 (Fig. 4b). If increased gene expression is associated with a poor prognosis, then its expression is increased in Cluster 2 and decreased in Cluster 1 (Fig. 4a). Conversely, if decreased expression is associated with poor prognosis, its expression is decreased in Cluster 2 and increased in Cluster 1 (Fig. 4b).

### Hub genes of the ccRCC cluster with the worst survival predict the response to TKIs

**Figure 5:**
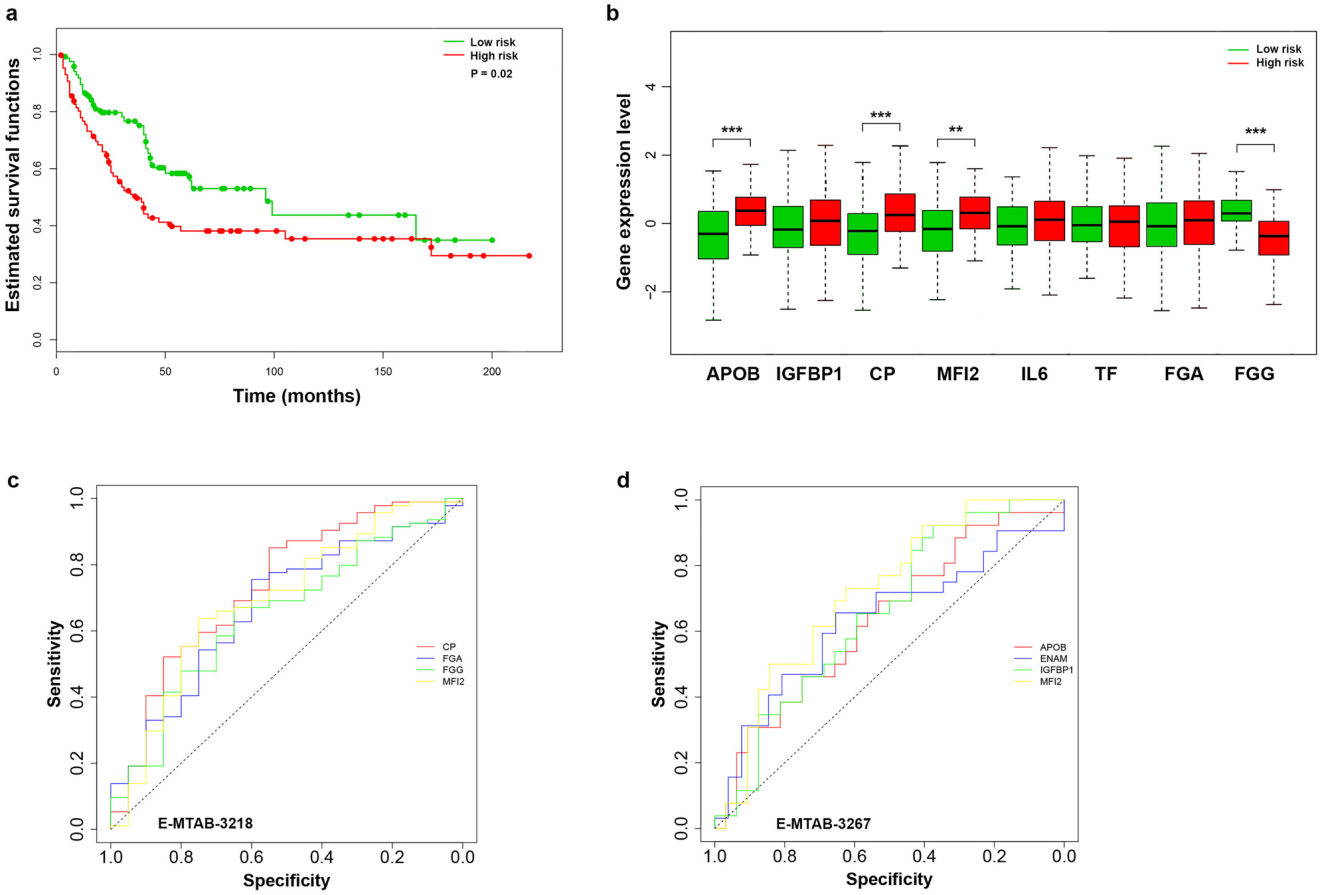
Analysis of the survival rate of the hub genes on an independent sample. Kaplan–Meier curve for the GSE3538 dataset (**a**). Expression of eight hub genes in high (red) and low risk (blue) groups (**b**). ***P < 0.001; **P < 0.001. ROC curves for four hub genes associated with response to nivolumab (**c**) and sunitinib (**d**).

To further check the association of Cluster 2 hub genes with poor survival in ccRCC in an independent sample, the GSE3538 dataset was used. Using multivariate Cox analysis, expression of a gene signature including *MFI2*, *APOB*, *IGFBP1*, *CP*, *FGA*, *FGG*, *IL6*, and *APOB* was shown to be associated with poor survival (*P* < 0.05, Fig. 5a). A statistically significant increase in expression in samples with a high risk was observed for the genes *APOB*, *CP* (*P* < 0.001), *MFI2* (*P* < 0.01) and a decrease in expression in samples with a high risk for the *FGG* gene (*P* < 0.001) (Fig. 5b). In addition, the datasets E-MTAB-3218 and E-MTAB-3267 showed that hub genes expression of Cluster 2 are associated with a response to TKIs nivolumab and sunitinib (*P* < 0.05) (Fig. 5c,d). The highest AUC and the most significant ROC *P*-value was found for *MFI2* with sunitinib (0.71, *P* < 0.001), and for *CP* (0.73, *P* < 0.001) with nivolumab.

### Association of the aggressive subtype of ccRCC with the TME

**Figure 6:**
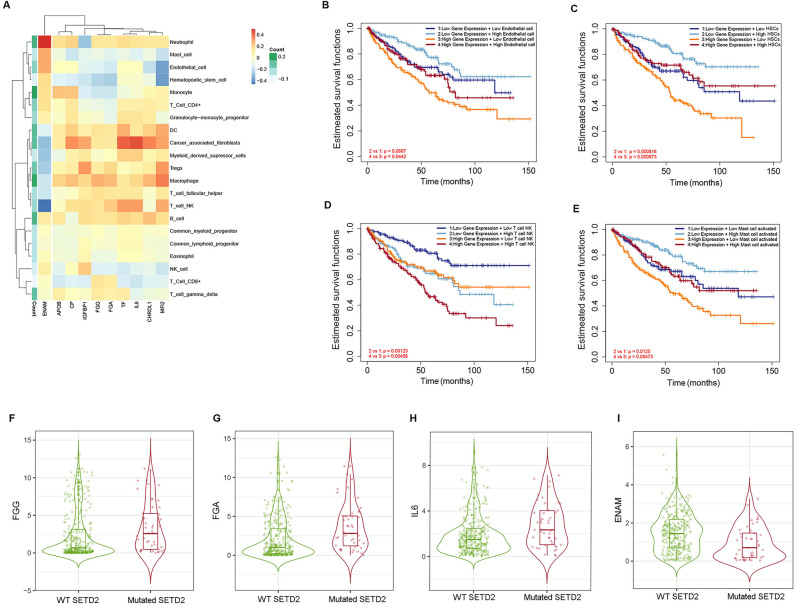
Association of the expression of the cluster 2 gene hub and the TME. The heat map shows the correlation coefficients of expression for 10 hubs of genes with the level of infiltration of immune cells in ccRCC (**A**). Cell color changes from blue to red depending on the Spearman's correlation coefficient. Clustering distance method was performed for rows by Pearson correlation. Survival curves for hub genes associated with levels of endothelial cell infiltration (**B**), hematopoietic stem cells (**C**), NK T cells (**D**) and mast cells (**E**). Correlation of the expression of *FGG* (**F**), *FGA* (**G**), *IL6* (**H**), and *ENAM* (**I**) with mutations in the *SETD2* gene (*P* < 0.001).

According to the TIMER2.0 database, the expression of the Cluster 2 hub genes correlates with the infiltration level of a number of cells in the TME in KIRC. In particular, with endothelial cells, HSCs, T cells NK, and mast cells. For the *ENAM* gene, there is an opposite correlation in comparison with other genes; in particular, a negative correlation with T cells NK and a positive correlation with Neutrophils (*P* < 0.05) (Fig. 6A). For *MFI2*, there is the strongest inverse correlation with endothelial cells and HSCs (*P* < 0.05) (Fig. 6A). In addition, according to the survival analysis, there is a negative correlation of 10 hub genes of Cluster 2 with endothelial cells and HSCs (Fig. 6B,C). At the same time, I found a positive correlation in survival time for natural killer T cells (T cells NK) and mast cells (Fig. 6C,D). We also found a significant positive correlation of mutations in the *SETD2* gene with the expression of the *FGG* (r_s_ = 0.55, *P* < 0.001), *FGA* (r_s_ = 0.78, *P* < 0.001), and *IL6* (r_s_ = 0.56, *P* < 0.001), and a negative correlation with the expression of *ENAM* (r_s_ = -0.67, *P* < 0.001, Fig. 6F–I). The frequency of the *SETD2* mutation in KIRC samples is about 12% (45/370).

## Discussion

Using data from patients with ccRCC from TCGA, this study identified specific genes for subtypes of ccRCC with the most and least favorable survival rates. The data obtained indicate the heterogeneity of clear cell renal carcinoma and are consistent with previous studies indicating two distinct molecular subtypes of ccRCC with different survival rates—ccA and ccB^10^. Ten hub genes have been identified for genes that are specifically expressed in this aggressive ccRCC subtype. According to gene set enrichment analysis, these genes are involved in vital cellular functions, such as post-translational protein modifications, endoplasmic reticulum, and insulin growth factor receptor signaling (Fig. S3b).

The revealed 10 hub genes associated with Cluster 1 and Cluster 2 represent targets for the development of targeted therapy for the corresponding ccRCC subtype. The involvement of some of the identified hub genes in the development and progression of ccRCC has already been reported earlier. In particular, increased *TF* expression and its effect on metastasis in ccRCC have been reported before^11^. As well as *IL6* has previously been reported as an early-stage immunologic prognostic factor for organ-confined ccRCC patients^12^. Recently, *IGFBP1* has been shown to regulate monocytes and play a vital role in the immune system of ccRCC tumors and enhances their progression^13^. According to the received data, a high level of T cells NK at a high expression of the Cluster 2 hub genes corresponds to poor survival, which indicates enhanced immunosuppression of the TME in this subtype of ccRCC (Fig. 6D).

It has also recently been shown that *ENAM* can suppress cell proliferation in ccRCC and significantly increase cytotoxic cells, CD56 NK cells, NK cells, and CD8 + T cells in the immune TME^14^. In this work, a correlation was revealed between the hub genes of the ccRCC subtype with poor prognosis and the immune cell infiltration cells. Negative correlation of *MFI2*, *FGA,* and *FGG* with endothelial cells and hematopoietic cells, which contribute to angiogenesis^15,16^. Apparently, this explains the poorer response to TKIs in samples with higher expression of *MFI2*, *CP*, *FGA*, *FGG,* and *APOB* (Fig. 5c,d). The negative correlation between the expression of the hub genes of Cluster 2 with mast cells and better survival at a high level of mast cells (Fig. 6E) is probably explained by the ability of mast cells to release heparin^17^.

Interestingly, *FGA* and *FGG* have been identified as specific markers for both clusters. An increase in the expression of these two genes, as well as *F2*, a coding prothrombin, is specific to poor prognostic Cluster 2 (Fig. 4b). The role of fibrinogen as a marker of poor prognosis and metastasis for kidney cancer has been shown previously^18^. Fibrinogen has an advantage as a prognostic marker due to its easier availability^19,20^. The key genes for Cluster 1 are associated with fibrinolysis and regulation of coagulation.

It is known that accumulations of lipids are observed in the cytoplasm of ccRCC tumor cells^21^, and the importance of changes in lipid metabolism in the development of ccRCC was studied previously^22^. Therefore, it is interesting that among the hub genes of the malignant subtype ccRCC, the *APOB* gene was also identified, encoding apolipoprotein B, which is involved in lipid metabolism and the main protein component of lipoproteins^23^.

In this work, it was revealed that mutations in the *SETD2* gene in ccRCC correlate with the expression of *FGG*, *FGA*, *IL6*, and *ENAM,* the hub-genes of Cluster 2 (Fig. 6F–I). Histone methyltransferase gene *SETD2* is frequently mutated and is a suppressor gene in ccRCC^24^. Recently, *SETD2* mutations have been shown to suppress autophagy in ccRCC^25^. In addition, mutations in *SETD2* are known to be one cause of sunitinib resistance in renal cell cancer^26^.

From the data obtained, it follows that increased coagulation is one of the key features of the malignant subtype of ccRCC. Apparently, in ccRCC, the hematogenous route of metastasis predominates in the lungs, bones, and liver^27^. In addition, according to the NDEx Biological Network Repository, *FGA*, *FGB*, and *FGG* are associated with positive regulation of heterotypic cell-cell adhesion (*P* < 0.001)^28^. Previous studies reported changes in the expression of *CP*, *FGA*, and *FGG* in metastatic ccRCC samples^29^. Therefore, the combined use of anticoagulants with conventional treatment can be useful in therapy of malignant forms of ccRCC. In particular, the safe use of sunitinib and dalteparin to treat metastatic forms of ccRCC has already been described^30^. There is also evidence that heparin, commonly used to treat thromboembolic events in cancer patients, contributes to their survival and has anti-metastatic activity^31^.

Thus, the subtypes of ccRCC associated with the least and most favorable prognosis and with better response to treatment were studied (Fig. S1). Hub genes and the processes in which they are involved were identified for each of the subtypes. The identified genes specific to both subtypes may be of interest for further study and development of new approaches to the treatment of different ccRCC subtypes.

## Methods

### Public data sources

Normalized data from 606 samples, their phenotypes including and clinical pathological characteristics and curated survival data, were downloaded from The Cancer Genome Atlas (TCGA) using the UCSC Xena Functional Genomics Browser^32^. The downloaded data included the RNA-Seq dataset (Illumina HiSeq platform, gene-level RSEM-normalized, log2 transformed). The GSE3538 database was taken as an independent verification sample, which includes microarray data from 177 conventional renal cell carcinoma samples. To analyze the response to tyrosine kinase inhibitors, I downloaded the E-MTAB-3218 database, including sequencing data of 114 biopsies from metastatic clear cell renal carcinoma before or after treatment with nivolumab, and E-MTAB-3267, including sequencing data from 59 patients with metastatic ccRCC treated with sunitinib.

### Clustering of ccRCC samples

Analysis of TCGA (The Cancer Genome Atlas) data performed using the Profiler of Multi-Omics data (PROMO) software^33^. This software, developed by Ron Shamir's lab, allows analyzing large genomic cancer datasets and their associated clinical information. The Cancer Genome Atlas Kidney Renal Clear Cell Carcinoma (TCGA-KIRC) data and their clinical characteristics were loaded into PROMO with further pre-processing and preparation for subsequent analysis. The integrity of the data was checked, and their distribution was characterized. Unsupervised clustering was carried out using the k-means method at k = 3 for samples and k = 5 for genes. The program algorithm was run 100 times. Identification of differentially expressed genes for individual clusters was carried out using the rank sum test. The change in expression at *P* < 0.05 was considered statistically significant. The predictive value of the identified sample clusters was studied by survival analysis using the Kaplan–Meier method and a log-rank test.

### Survival and ROC analysis

Survival analysis was performed using the Gene Expression Profile Interactive Analysis GEPIA tool^34^. Survival plots were constructed using the Kaplan–Meier method with cut-off by medians. The difference in the samples of low and high expression is considered through the logrank test. *P-*values < 0.05 were considered statistically significant. To visualize differentially expressed genes in ccRCC clusters and construct the histogram of gene expression distribution, I used the ImageGP platform (http://www.ehbio.com/ImageGP). The construction of survival curves by the Kaplan–Meier method for the control dataset GSE3538, including RNA-Seq data of 177 ccRCC patients, was carried out using the SurvExpress tool, which allows analyzing the prognostic value of multiple genes as a signature^35^. The ROC plotter tool^36^ was used to analyze the predictive ability of genes for response to TKIs. Multivariate Cox regression was used to construct the prognostic risk models.

### Building networks of protein–protein interactions

The construction of networks of protein–protein interactions carried out using the Cytoscape program. The interaction data was downloaded from the STRING database of known and predicted protein–protein interactions, version 11^37^. Differentially expressed genes in TCGA samples were identified using the GEPIA tool with ANOVA test and q-value cutoff = 0.05. The identification of hub genes was carried out using the CytoHubba plugin with the topological analysis method Maximal Clique Centrality (MCC)^8^. Gene set enrichment analysis (GSEA) was performed for identification of pathways associated with hub genes^38^. Visualization of networks of interactions was carried out using Cytoscape 3.7.1 software^39^.

### Analysis of the correlation of gene expression with the TME

The Tumor Immune Estimation Resource (TIMER2.0) resource was used to analyze the relationship between gene expression and the level of immune-cell infiltration in the ccRCC TME^40^. TIMER2.0 allows us to estimate the immune infiltration levels for TCGA using six state-of-the-art algorithms, including TIMER, xCell, MCP-counter, CIBERSORT, EPIC, and quanTIseq. In addition, using TIMER2.0, I searched for the correlation of gene expression levels with the status of non-synonymous somatic mutations in the most frequently mutated genes in ccRCC.

### Statement

TCGA and GEO belong to public databases. The patients involved in the database provided ethical approval. Users can download relevant data for free for research and publish relevant articles. This study is based on open-source data, so there are no ethical issues.

## Supplementary Information

Supplementary Information.

## Data Availability

The datasets analyzed during the current study are available from the corresponding author on reasonable request. All methods were carried out in accordance with relevant guidelines and regulations.

## Abbreviations

ccRCC: Clear cell renal carcinoma
PPI: Protein–protein interactions
DEGs: Differentially expressed genes
KIRC: Kidney renal clear cell carcinoma
HSCs: Hematopoietic stem cells
TKIs: Tyrosine kinase inhibitors
TME: Tumor microenvironment
