# Supplementary material for: Identification of key genes of the ccRCC subtype with poor prognosis
Source: Sci Rep. 2022 Aug 26;12:14588. doi: 10.1038/s41598-022-18620-y (PMC9418309; doi:10.1038/s41598-022-18620-y)
Supplement: Supplementary file 1 — Supplementary Information. [file 41598_2022_18620_MOESM1_ESM.pdf]

Supplementary material

Figures S1 to S4.

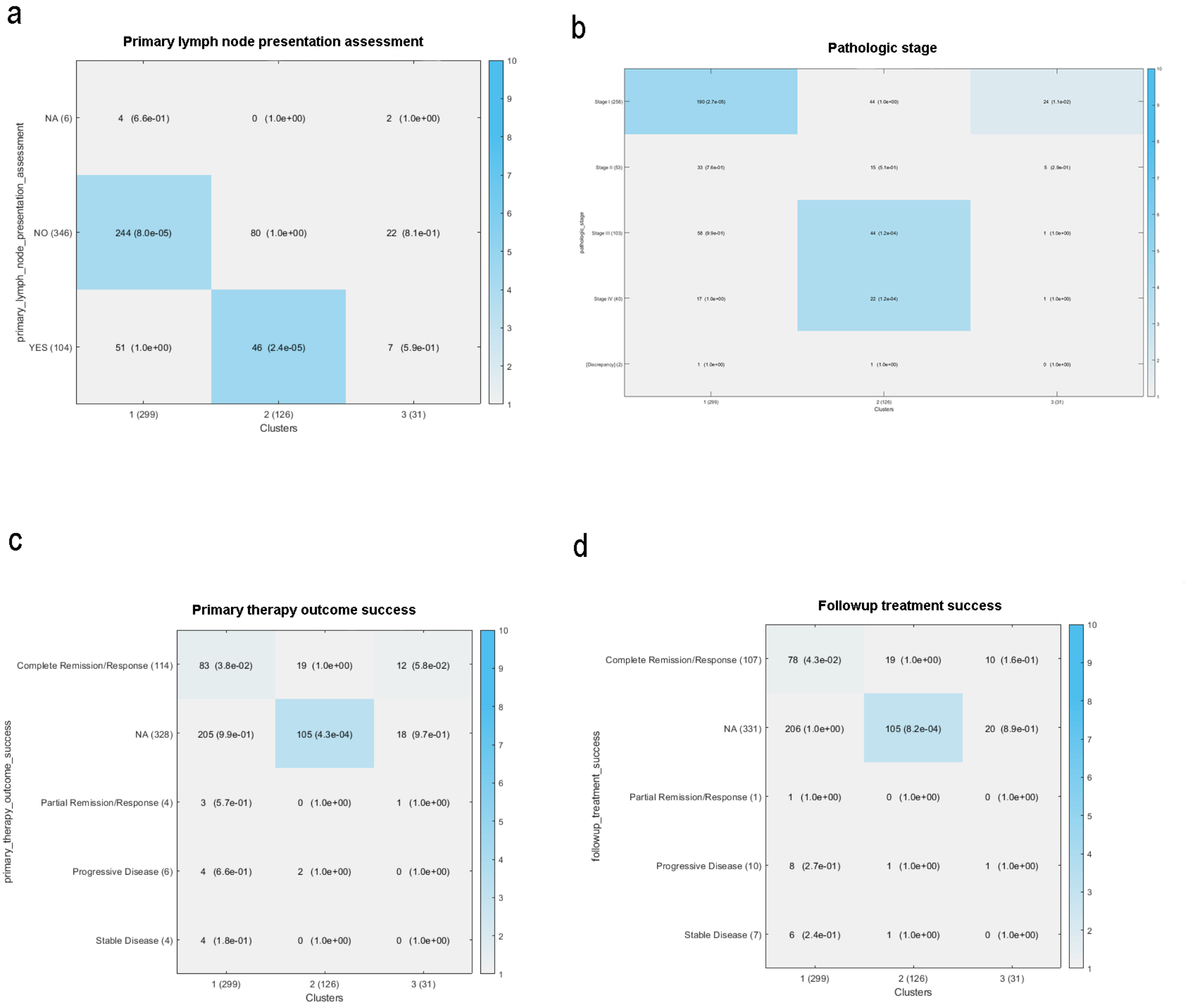

Figure S1. Distribution of samples with primary lymph nodes (a), pathologic stage (b), primary therapy outcome success (c) and follow up treatment success (d) depending on the cluster. The blue gradient increases towards a more significant *P*-value. The difference was considered significant at the $P < 0.05$.

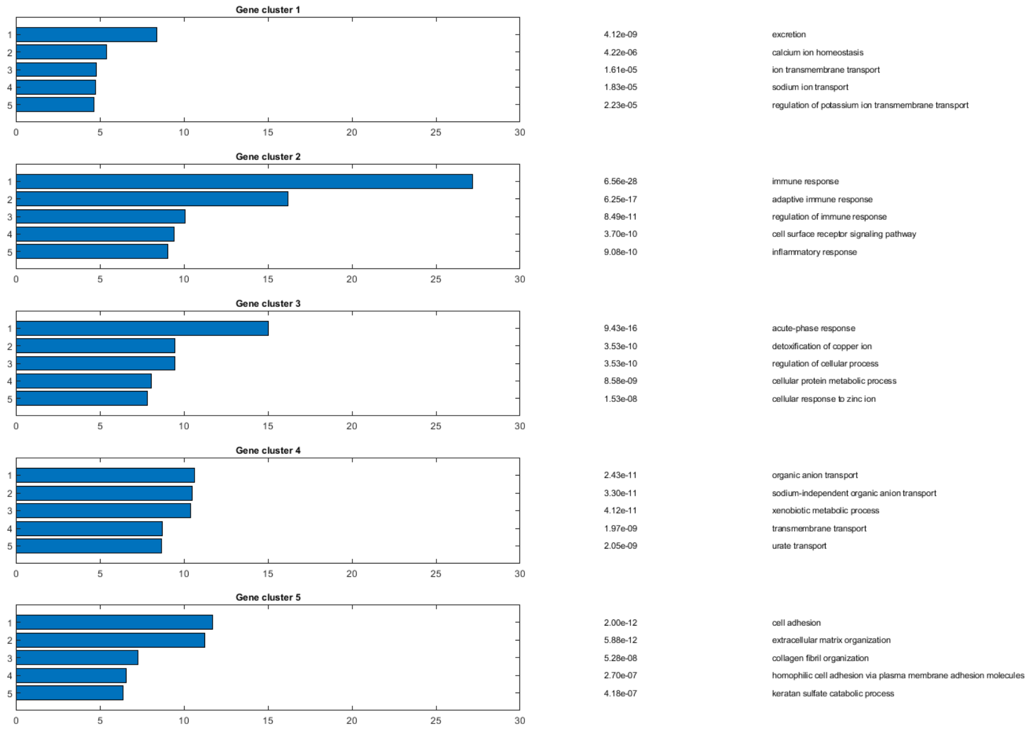

Figure. S2. Gene Ontology analysis for five revealed gene clusters.

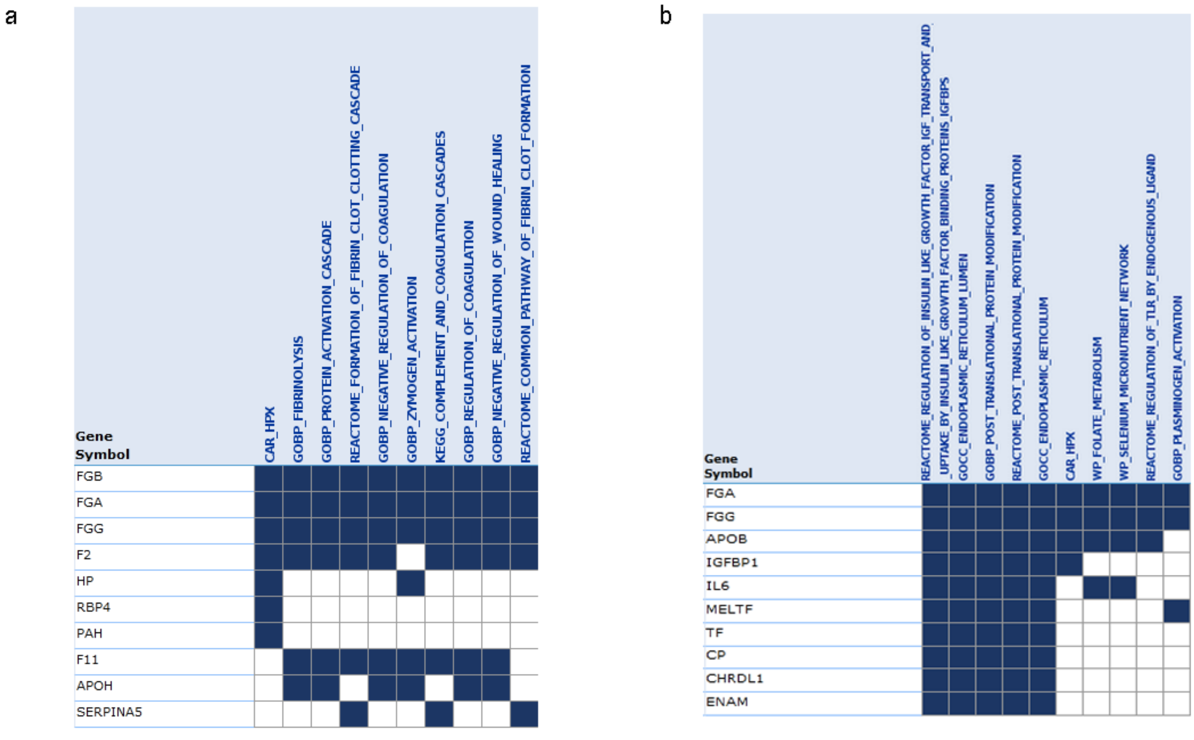

Figure. S3. Gene Set Enrichment Analysis (GSEA) for hub genes of Cluster 1 (a) and Cluster 2 (b).

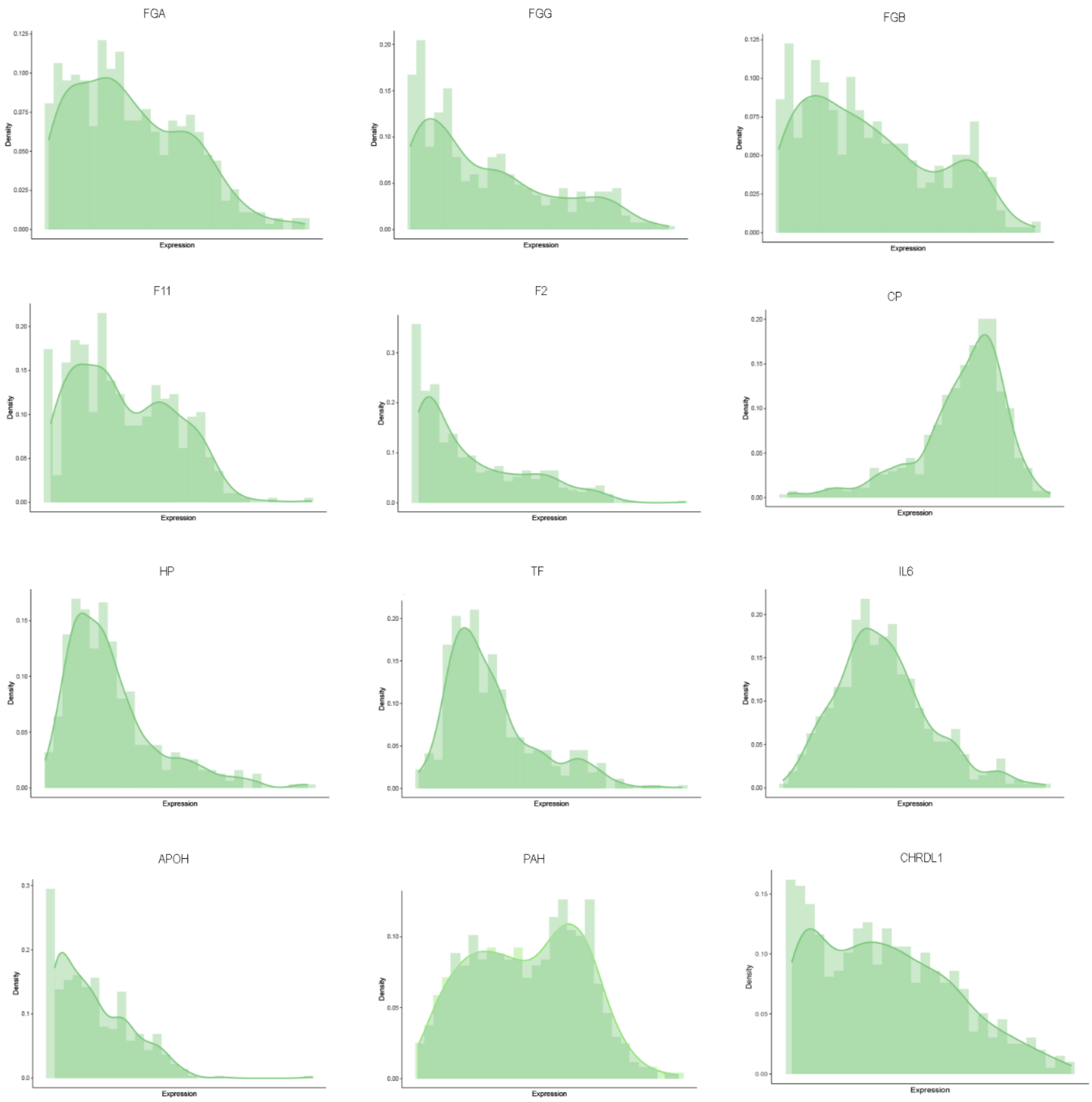

Figure S4. **Expression distribution histograms for hub genes.**
